# Effect of bovine milk fat-based infant formulae on microbiota, metabolites and stool parameters in healthy term infants in a randomized, crossover, placebo-controlled trial

**DOI:** 10.1186/s40795-022-00575-y

**Published:** 2022-08-29

**Authors:** Ellen Looijesteijn, Rutger W. W. Brouwer, Ruud J. W. Schoemaker, Laurien H. Ulfman, Stephanie L. Ham, Prescilla Jeurink, Eva Karaglani, Wilfred F. J. van IJcken, Yannis Manios

**Affiliations:** 1 FrieslandCampina, Amersfoort, The Netherlands; 2 Department of Cell Biology, Erasmus MC, Rotterdam, The Netherlands; 3 Center for Biomics, Erasmus MC, Rotterdam, The Netherlands; 4 Metabolon Inc, Morrisville, NC USA; 5 Department of Nutrition and Dietetics, School of Health Science and Education, Harokopio University, Athens, Greece; 6 Institute of Agri-Food and Life Sciences, Hellenic Mediterranean University Research Centre, Heraklion, Greece

**Keywords:** Milk fat, Microbiota, Deep sequencing, Faecal metabolites, SN-2-palmitate, Calcium excretion, Stool consistency, Faecal fatty acid soaps

## Abstract

**Background:**

Natural enrichment of sn-2 palmitate content of infant formulae by using bovine milk fat is known to reduce formation of faecal fatty acid soaps and to improve stool consistency, but effects on gut microbiota composition are unknown. The purpose of this study was to test the influence of milk fat-based formula high in sn-2 palmitate on the infants’ gut microbiota composition and to confirm the beneficial effects of the formula on formation of faecal fatty acid soaps and stool consistency.

**Methods:**

Twenty-two healthy term, formula-fed infants were enrolled in a single-blinded randomized, crossover, placebo-controlled trial. After a 2-week run-in period, infants received either a 50% milk fat-based formula containing 39% sn-2 palmitate (MF) or a vegetable fat-based formula (VF) containing 10% sn-2 palmitate in a 2 × 2-week crossover design. Faecal microbiota composition was the primary outcome of the study. Other outcomes included faecal fatty acid soap excretion, calcium excretion, gut comfort parameters and faecal metabolites.

**Results:**

Microbiota analysis showed that bifidobacteria dominated the gut microbiota of most infants. Neither alpha- nor beta-diversity was significantly influenced by the intervention. Also, abundance of metabolic pathways was independent of the intervention. The MF formula resulted in significantly lower faecal levels of palmitic acid soap (*p* = 0.0002) and total fatty acid soaps (*p* = 0.0001) than the VF formula. Additionally, calcium excretion and palmitic acid concentration were significantly (*p* = 0.0335) lower in stool samples after MF intervention. Furthermore, a significant physiological effect on softer stools was observed in the MF intervention compared to the VF intervention (*p* = 0.02).

Of the 870 measured faecal metabolites, 190 were significantly different after MF and VF intervention (FDR corrected *p* < 0.05). Most of these were found at higher levels after MF intervention, potentially indicative of the complex structure of milk fat. Metabolites with more than twofold change between interventions were mostly lipid-derived and included several milk fat-specific fatty acids.

**Conclusions:**

Replacing part of the vegetable fat in infant formula with bovine milk fat with high sn-2 palmitate levels did not change the microbiota composition, although a reduction in faecal palmitate soaps, total fatty acid soaps and calcium excretion while improving stool consistency in the MF intervention was confirmed. In addition, 190 faecal metabolites were significantly different, many related to the fat source.

**Trial registration:**

Netherlands Trial Registry Identifier: NL7815 19/06/2019.

**Supplementary Information:**

The online version contains supplementary material available at 10.1186/s40795-022-00575-y.

## Background

Human milk is designed by nature and is the best nutrition for healthy growth and development of babies. The fat fraction of human milk is particularly important for a developing infant. It delivers a large part of the required energy as well as essential fatty acids, fat-soluble vitamins and other bioactive components, such as phospholipids and cholesterol [1]. Most of the human milk fat fraction comprises triglycerides with a specific distribution of the fatty acids over the glycerol backbone that facilitates absorption. Palmitic acid (C16:0) is the most common long-chain saturated fatty acid (LCSFA) in human milk, and 70–88% of all C16:0 is esterified at the sn-2 position [2]. At this position, LCSFAs are easily absorbed during digestion in the form of monoglycerides. In contrast, LCSFAs esterified at the sn-1 or sn-3 positions, liberated from the glycerol backbone by pancreatic lipases during digestion, are poorly absorbed because of their low water solubility. Consequently, complexes with calcium can be formed, resulting in calcium soaps that are excreted in the faeces [1].

Vegetable fat blends are often used as fat sources in infant formulae (IF). However, commonly used fat blends contain only 10–20% C16:0 at the sn-2 position. The remaining 80–90% C16:0 esterified at the sn-1 and sn-3 positions in triglycerides increases the risk of soap formation. In addition to reduction of fat and calcium absorption, this is undesired because of the association with hard stools and constipation in infants [2]. In order to reduce formation of fatty acid soaps, fat blends used in IF should preferably have more C16:0 positioned at the sn-2 position of the triglyceride instead of at sn-1 and sn-3 positions. Bovine milk fat composition contains 40–45% of C16:0 at the sn-2 position which is far higher than most vegetable fats [2, 3]. Recently, the similarity index has been developed as a tool to design the optimal composition of fat blends [4]. Indeed, an infant formula with a mixed fat blend of selected vegetable oils and bovine milk fat (50%) reduced formation of faecal fatty acid soaps and improved stool consistency compared to a standard IF with 100% vegetable fat in a recent clinical study [5]. In addition to an effect on stool consistency, faecal soaps might also influence gut microbiota composition and activity. In vitro, calcium palmitate inhibited the growth of the beneficial bacterium *Faecalibacterium prausnitzii* and several *Bifidobacterium* species [6]. Furthermore, several clinical trials demonstrated higher faecal concentrations of bifidobacteria in infants fed with IF rich in sn-2 palmitate than in infants fed with IF low in sn-2 palmitate [7–9]. In these trials, vegetable fat in the form of palm oil was used, which was chemically structured (inter-esterified) to enrich the sn-2 palmitate content of the IF.

The effect of IF differing in sn-2 content by partly replacing the fat source with bovine milk fat on gut microbiota composition and activity is largely unknown. The objective of the current study was to compare IF with a mixed fat blend of 50% vegetable oils and 50% bovine milk fat, containing 39% sn2-palmitate, with a standard formula with the same total fat content but originating from a 100% vegetable fat blend containing 10.1% sn2-palmitate. The primary outcome parameter was faecal microbiota composition, the secondary outcomes were faecal palmitic acid soap, faecal free palmitic acid, total faecal fatty acid (soaps), faecal calcium excretion, stool characteristics and gut comfort and, additional outcomes included faecal metabolites.

## Methods

### Study design and population

**Fig. 1:**
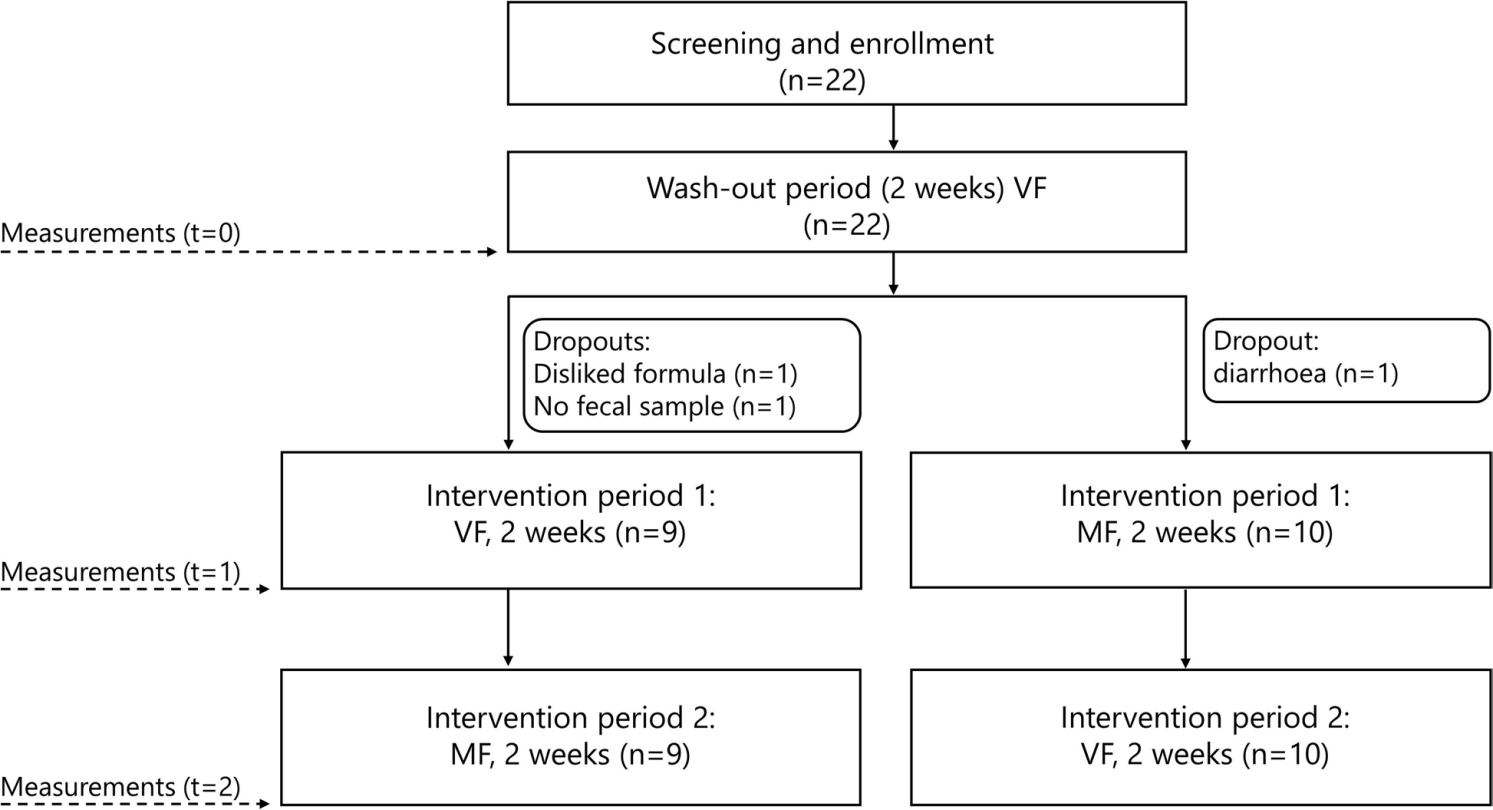
Study flowchart. *VF* standard formula with 100% vegetable fat source, *MF* test formula with 50% milk fat

The clinical trial was a single-blinded, crossover, randomized, placebo-controlled study conducted with healthy, full-term, exclusively IF-fed infants. The total duration of the study was 6 weeks, including a 2-week run-in period during which the infants consumed standard IF with a 100% vegetable fat source (VF). Infants were enrolled in the study during routine visits to the study pediatricians. Upon inclusion in the study, the randomization was performed centrally, at Harokopio University, by a designated research assistant who assigned the participants to the treatment arms. Upon enrolment, the infants were alternately allocated to consume the VF formula or a test formula with 50% milk fat and 50% vegetable fat (MF) in a 2 × 2 week crossover design after the run-in period (Fig. 1). The parents, paediatricians and statisticians were blinded to the IF. The study was approved by the ethics committee of Harokopio University (Athens, Greece) and was conducted between May 2019 (first subject in) and November 2019 (last subject out) in Athens, Greece. The trial was conducted in agreement with the International Conference on Harmonisation guidelines on Good Clinical Practice and was registered at the Dutch Trial Register (trialregister.nl) as NL7815.

Infants were recruited during routine visits to their private paediatricians. They were enrolled at 9–16 weeks of age. Infants were eligible for inclusion when they were healthy, full-term (gestational age ≥ 37 weeks) with a birth weight between the 10th and 90th percentiles, and exclusively formula-fed at least two weeks before enrolment and during the entire study. Complementary feeding was initiated only after the endpoint measurements. Exclusion criteria included severe acquired or congenital diseases, mental or physical disorders, any symptoms of allergy, parents or siblings with documented cow’s milk allergy, use of probiotics, antibiotics or other medication that treat or cause gastrointestinal symptoms, use of medication known or suspected to affect fat digestion, absorption and/or metabolism, such as nutritional supplements, suppositories, medication that may suppress or neutralize gastric acid secretion and gut motility and participation in another clinical trial. Written informed parental consent was obtained for each infant from both parents. In total, 22 infants were enrolled in the study.

Primary outcome of the study was defined as a difference in intestinal microbiota composition between MF and VF intervention. Secondary outcomes of the study were defined as a decrease in faecal excretion of fatty acid soaps and calcium by the MF intervention and improved gut comfort parameters in the MF intervention.

### Study formulae and formula consumption

Two different IF were compared in the study: standard IF with a 100% vegetable fat source (VF) and test formula with 50% milk fat and 50% vegetable fat (MF). The nutritional composition of the two study products was similar regarding macronutrients, with the only difference being their fatty acid profiles and percentage of sn-2 palmitate (Table 1). Both IF complied with the compositional requirements laid down in Directive 2006/141/EC. The products were identical to IF used in a previously published study [5]. The products were produced by FrieslandCampina in the Netherlands and were packed in similar tins of 400 g. Instructions for the parents/caregivers on daily volume of formula intake were given on the labels of the tins. The parents/caregivers recorded formula consumption (timing, frequency and exact volume) in a diary during the last three consecutive days of the run-in period and the two intervention periods. Additionally, the study personnel collected all formula tins to monitor compliance and formula consumption.

**Table 1:** Macronutrient, fatty acid and calcium composition of infant formulae

Nutrient/Ingredient	VF	MF
Energy (kcal/100 ml)	66	66
Intact protein (g/100 ml)	1.4	1.4
Carbohydrates (g/100 ml)	7.0	7.1
Galacto-oligosaccharides (g/100 ml)	0.27	0.27
Fat (g/100 ml)	3.5	3.5
*Fatty acids; mol% of TAGs*		
C12:0; Lauric acid	10.4	6.0
C14:0; Myristic acid	3.9	7.4
C16:0; Palmitic acid	24.9	18.9
C18:0; Stearic acid	3.4	5.2
C18:1; Oleic acid	39.0	36.9
C18:2; Linoleic acid	12.7	11.7
C18:3; a-Linolenic	1.8	1.5
C20:0; Arachidic acid	0.3	0.2
% C16:0 in sn-2 position	10.1	39
Calcium (mg/100 ml)	56	53

*VF* standard formula with 100% vegetable fat source, *MF* test formula with 50% milk fat

### Stool collection

Parents collected stool samples during the last three days (days 12–14) of the run-in period and each of the two intervention periods until ~ 30 g was collected in total. Each freshly passed stool was placed in a faecal tube collector, kept in a ziplock amber plastic bag and then immediately stored in the home freezer. The pooled faecal samples of 30 g of each infant per period were kept frozen until analysis and transported in dry ice. The fat composition and calcium concentration of the samples were determined by Eurofins Laboratories, Madison, Wisconsin, USA, as described previously [5].

### DNA isolation, library preparation and sequencing

DNA was isolated from lyophilized faecal material using the QIAamp Fast DNA Stool Mini Kit (QIAGEN, Venlo, The Netherlands) according to the manufacturer’s instructions. Isolated DNA samples were quantified using the Quantit method (Thermo Fisher Scientific). Fifty nanograms of DNA was used to generate dual-indexed sequencing libraries according to the DNA Flex method (Illumina) using 5 PCR amplification cycles. The resulting libraries were sequenced on Illumina HiSeq2500 sequencers (Illumina) according to the manufacturer’s instructions. Paired-end reads were generated of 300 base pairs in length using an in-house developed sequencing protocol. Between 20–24 M reads were generated per sample.

Illumina adapters were removed from the paired-end reads using AdapterTrimmer (https://github.com/erasmus-center-for-biomics/AdapterTrimmer), and the remainder was processed using MetaPhlan version 3.0.6 using the CHOCOPhlAn reference (version 201,901) [10]. This analysis identified the microbial composition of the samples up to the species level and served as input for the downstream analysis. Downstream analysis was performed using the phyloseq package in R (version 4.1.1)[11]. The relative abundances of metabolic pathways were determined using the HUMAnN3 workflow[10].

### Metabolomics

Metabolon (Morrisville, North Carolina, USA) performed the metabolite analysis using the HD4 Platform. Lyophilized faecal samples were extracted with methanol to precipitate protein and dissociate small molecules bound to protein or trapped in the precipitated protein matrix, followed by centrifugation to recover chemically diverse metabolites. The resulting extract was divided into fractions. These were used for analysis by two separate reverse phase (RP)/UPLC-MS/MS methods using positive ion mode electrospray ionization (ESI), for analysis by RP/UPLC-MS/MS using negative ion mode ESI and for analysis by HILIC/UPLC-MS/MS using negative ion mode ESI. Metabolon’s peak identification software was used to match ions to a library of standards for metabolite identification and for metabolite quantitation by peak area integration.

### Gut comfort, stool consistency and anthropometric measurements

During home visits after the run-in period and the two intervention periods, research assistants completed the "Questionnaire on Paediatric Gastrointestinal Symptoms" (QPGS-RIII infant/toddler) [12] together with the parents. This assessed overall gut comfort and the incidence of minor digestive issues, such as vomiting, regurgitation, colic, constipation, diarrhoea and crying episodes. Parents used the "Amsterdam Infant Stool Scale" (AISS) [13] to assess consistency (four categories: watery, soft, formed and hard), amount/volume (smear to more than 50% of the nappy’s surface) and colour (six categories) of stools during the run-in period and both intervention periods. This was done during the same three days as stool collection. A detailed description of methods to determine QPGS-RIII and AISS, can be found in Additional file 1. Infant body weight and length were measured during scheduled visits at study locations at the end of the three two-week periods.

### Safety

Adverse events (AEs) and serious adverse events (SAEs) were recorded throughout the study and monitored by an independent pediatrician. No code-break requests occurred for AEs or SAEs throughout the study. One adverse event was reported during the study, diarrhea during the run-in period, and this infant discontinued the study (see Fig. 1).

### Statistical analysis

Statistical analyses on the primary outcome, microbial composition, included alpha and beta diversity, MDS visualisation, as well as ANOVA and Kruskal–Wallis rank sum tests. To determine whether the interventions affected the microbial diversity in the infants, both the Shannon alpha diversity and Bray–Curtis beta diversity were determined via the phyloseq R package [11] using standard parameters. Multidimensional scaling (MDS) was used to visualize the beta diversity. Subsequently, ANOVA via the aov function in R was used to determine associations between the axes along the MDS plot and registered clinical factors of the subjects. Clustering was performed on the Bray–Curtis beta-diversity using hierarchical clustering with the ward. D2 linkage method as available in R. The microbes differentially present in the clusters were determined using Kruskal–Wallis rank sum tests. The analysis scripts are available on GitHub (https://github.com/erasmus-center-for-biomics/bamboo-study-R). In addition, the microbes were also analyzed in R according to the methodology described below for the biochemical measurements and clinical outcomes. Pathways occurrence expressed in reads-per-million were normalized across the samples using a z-score transformation. Pathways were ordered using ward.D2 hierarchical clustering. Wilcoxon tests were performed to determine whether pathways were differentially abundant upon milk-fat and vegetable-fat based product intake.

The biochemical measurements of the faecal samples and clinical outcomes were analysed by 4Pharma Ltd. (Finland) using SAS® version 9.4 for Windows (SAS Institute Inc., Cary, NC, USA). For all endpoints, a *p*-value less than 0.05 was considered statistically significant.

An ANOVA model appropriate for a 2 × 2 crossover design was used to assess mean differences between treatments for the different parameters. When the normality assumption was not met, variables were log-transformed, or the Wilcoxon signed-rank test was applied. The statistical model included treatment, sequence and period as fixed effects and subject (sequence) and residual error terms as random effects. All statistical tests were two-sided and performed with α = 0.05. Missing values were not imputed in the analyses. Demographics, birth history, feeding habits, family socio-demographics, medical history, concomitant treatments, and baseline QPGS-RIII questionnaire were summarized using descriptive statistics for continuous variables and counts and percentages for categorical variables. Ordinal variables were assessed with a repeated measures cumulative logit model.

Metabolon (Morrisville, North Carolina, USA) used crossover ANOVA to statistically analyse the metabolite data. For all analyses, missing values, if any, were imputed with the observed minimum for that particular compound. The statistical analyses were performed on natural log-transformed data. To control for false discovery rate (FDR), FDR corrected *p*-values (*q*-values) were determined [14]. To determine which metabolites made the largest contribution to the classification, RFA was performed [15] based on the computed “Mean Decrease Accuracy” (MDA) using the "randomForest" R package [16]. The MDA was determined by randomly permuting a metabolite, running the observed values through the trees, and then reassessing the prediction accuracy. The values of the metabolite are permuted for the out-of-bag (OOB) samples for each tree. The classification accuracy for each of these trees was compared to the original classification error. These differences were averaged across all trees and re-scaled based on the standard deviation of these (and number of trees).

## Results

### Study population

Twenty-two infants were enrolled in the study, of which 19 completed the study. Three infants discontinued during/after the run-in period because of either diarrhoea (1), disliking the formula (1) or failure to deliver data and samples (1) (Fig. 1). Complete datasets were obtained for 16 infants, 7 in the VF-MF group and 9 in the MF-VF group. For one infant in each of the groups, no faecal samples were obtained in the second intervention period, and for that reason, faecal samples of these infants were not analysed. For another infant in the VF-MF group, questionnaires and biochemical analysis of the faecal samples were lacking. Additional file 2, Supplementary Table 1 gives an overview of IF allocation and availability of data per subject.

The baseline and family characteristics of the infants are described in Table 2. Weight at birth, gestational age and infants’ age and weight at inclusion were similar among the groups.

**Table 2:** Baseline and family characteristics of the study subjects

	MF-VF (*n *= 10)	VF-MF (*n* = 9)
**Gender, *n*** **(%)**		
Female	5 (50.0)	4 (44.4)
Male	5 (50.0)	5 (55.6)
**Age at screening, days**		
Mean (SD)	92.0 (11.6)	89.4 (14.6)
Median	89	84
Min–max	70–112	67–111
**Ethnicity, *n*** **(%)**		
Albanian	1 (10.0)	1 (11.1)
Greek	9 (90.0)	8 (89.9)
**Length at birth, cm**		
Mean (SD)	49.4 (2.0)	50.2 (1.5)
Median	50.0	50.0
Min–max	44.0–51.0	49.0–54.0
**Weight at birth, g**		
Mean (SD)	3063.0 (372.8)	2935.0 (265.0)
Median	3150.0	2910.0
Min–max	2500–3480	2500–3455
**Gestational age, weeks**		
Mean (SD)	37.7 (0.8)	38.6 (0.5)
Median	37.5	39.0
Min–max	37–39	38–39
**Mode of delivery, *n*** **(%)**		
C-section	8 (80.0)	6 (66.7)
Natural delivery	2 (20.0)	3 (33.3)
**Length at screening, cm**		
Mean (SD)	62.05 (2.2)	62.1 (3.1)
Median	61.5	61.5
Min–max	59.0–65.3	58–68
**Weight at screening, g**		
Mean (SD)	5963.7 (601.2)	5827.1 (945.9)
Median	5958.5	6180
Min–max	4860.0–7083.0	4130–7200

*VF* standard formula with 100% vegetable fat source, *MF* test formula with 50% milk fat, *SD* Standard deviation. *MF-VF* crossover group with MF first, *VF-MF* crossover group with VF first

### Formula consumption and anthropometric data

The average weekly milk intake and the infants’ weight and length development during the study were similar for the MF-VF and VF-MF groups (Additional file 2, Supplementary Table 2).

### Microbiota composition and pathway analysis

**Fig. 2:**
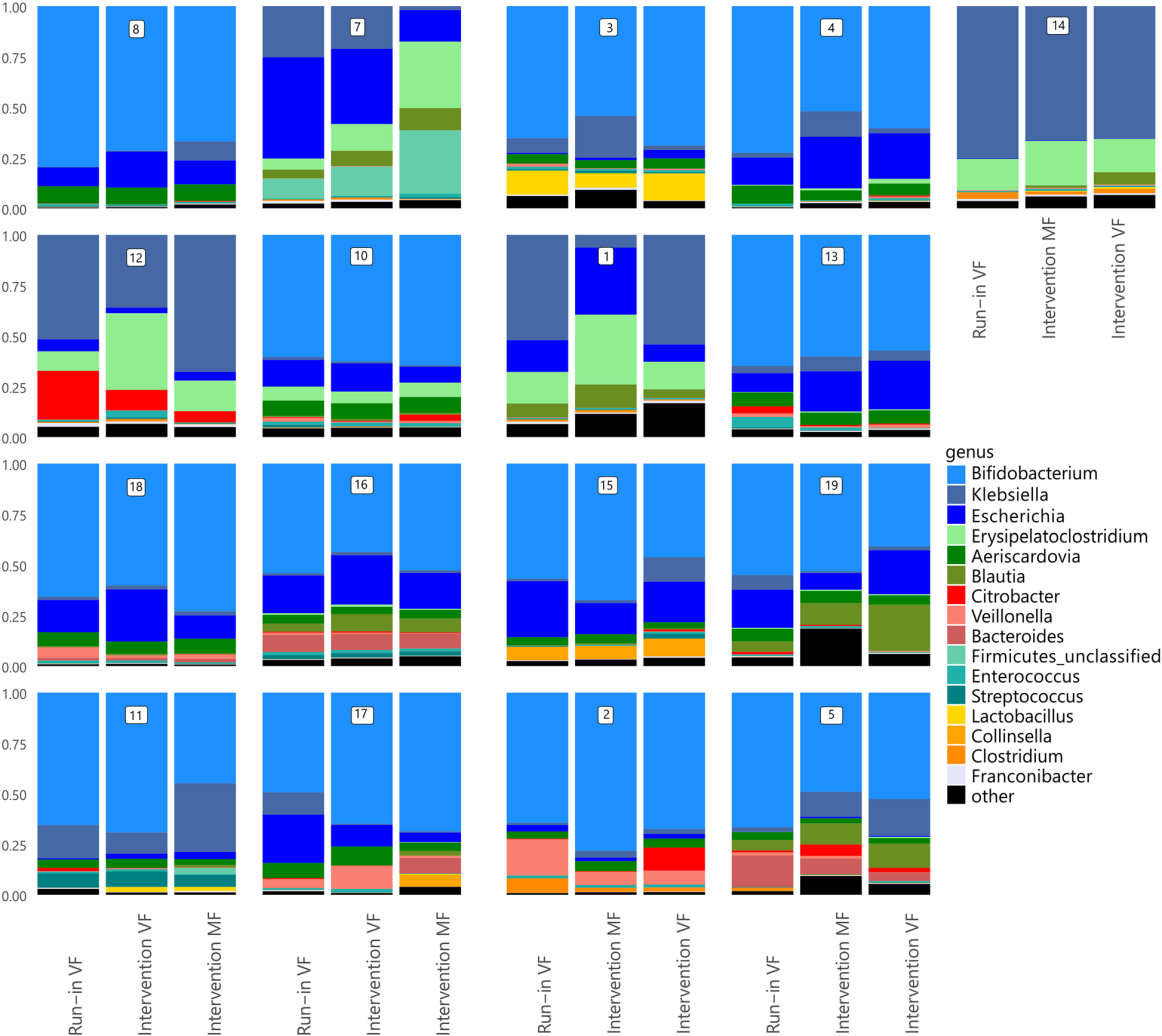
Relative abundance of bacterial genera in the three faecal samples for each of the subjects. *VF* standard formula with 100% vegetable fat source, *MF* test formula with 50% milk fat

The most abundant genus in the faecal microbial profiles for most infants (13 out of 17 infants, 76%) was *Bifidobacterium* which is in line with previous reports [17, 18]. The most abundant genera in faeces of the other infants were *Klebsiella* (3 children) and *Escherichia* (1 child). Through the intervention, changes in the microbial profiles were observed. However, the variation in the microbiome between the infants was larger than the effect of the intervention (Fig. 2 and Additional file 2, Supplementary Fig. 1). Furthermore, no specific clades were directly and significantly affected by the interventions performed in this study. We performed a basic univariate analysis on each separate type of the 250 different species, where only *Enterococcus faecalis* was significant before multiple testing (*p* = 0.018), though not after multiple testing correction, due to the high number of species tested and relatively low sample size.

To determine whether the interventions affected the microbial diversity in the infants, both the Shannon alpha diversities and Bray–Curtis beta diversities were determined; however, neither of these methods showed an intervention-specific effect.

**Fig. 3:**
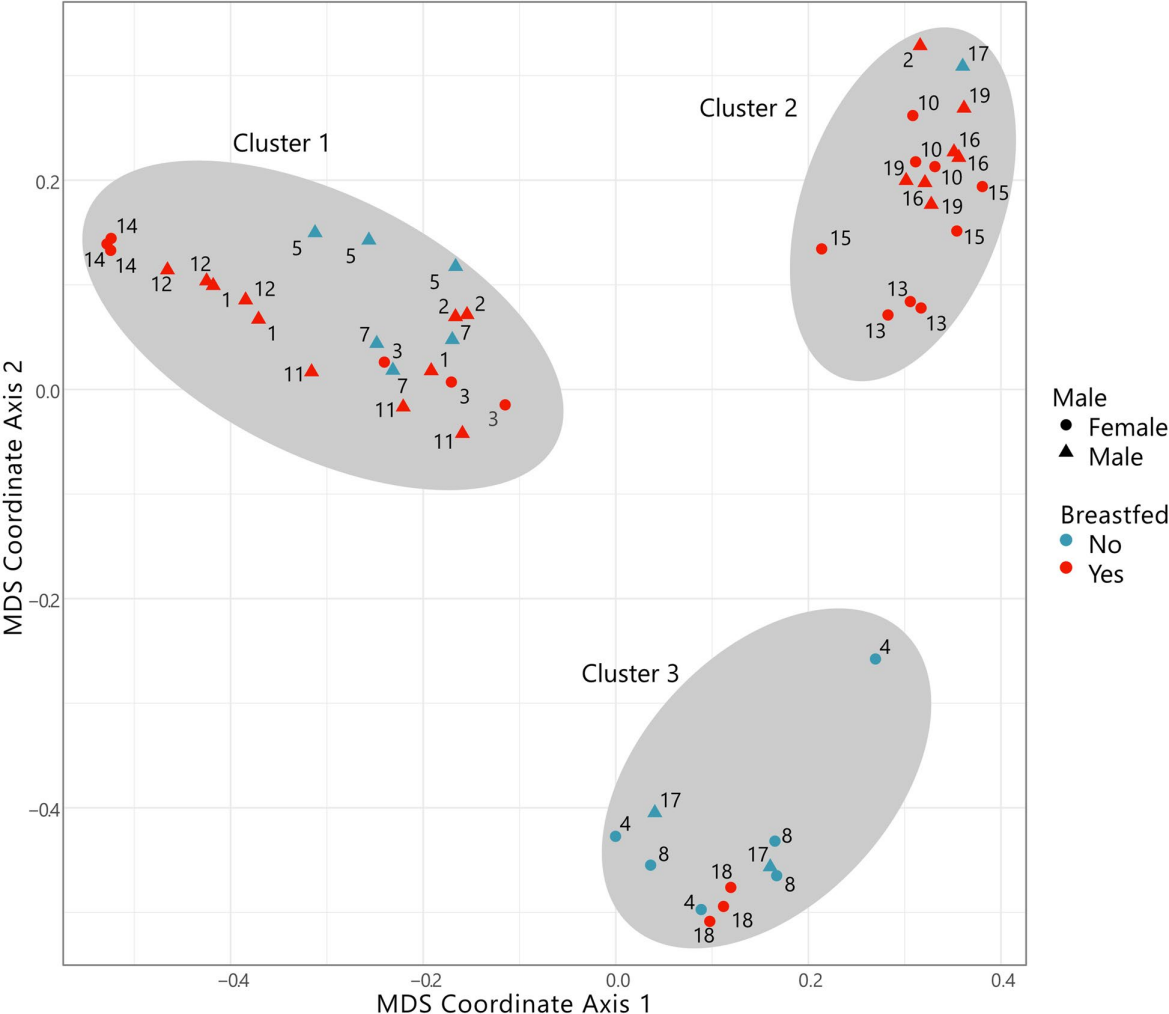
Multi-dimensional scaling (MDS) plot based on Bray–Curtis dissimilarity measures of the faecal microbial species

Multidimensional scaling (MDS) analysis identified three beta diverse distinct clusters, also found with hierarchical clustering (Fig. 3). These sample clusters do not correspond to the time of sampling or interventions. With few exceptions, the samples from singular infants localize together. Of the abundant microbes, the relative proportion in the samples of the genera *Aeriscardovia,* consisting only of the species *Aeriscardovia aeriphila* that was formerly known as *Bifidobacterium aerophilum* (adjusted *p*-value 2.4E-3) and *Klebsiella* (adjusted *p*-value 9.4E-6), primarily differentiate cluster 1 from 2 and 3. In addition, cluster 2 can be differentiated from clusters 1 and 3 by their, albeit very low (< 0.08%), relative abundance of *Thermoleophilum* (adjusted *p*-value 8.1E-6), a genus of the phylum Actinobacteria. In total, the relative abundances of 16 genera were significantly different between the 3 clusters (Additional file 2, Supplementary Fig. 2). Using Pearson's chi square tests, the clusters were compared to infant-specific categorical clinical parameters. Clusters were significantly associated with gender, prior breastfeeding and bowel frequency (Table 3). Cluster 3 was enriched for females and non-breastfed infants, and cluster 2 was enriched for priorly breastfed infants.

**Table 3:** Clinical parameters significantly associated with beta diversity

Parameter	*p*-value	Adjusted *p*-value (FDR)
Gender ^p^	0.007	**0.034**
Prior Breastfeeding ^p^	0.001	**0.009**
Mode of delivery ^p^	0.852	0.852
Bowel frequency ^p^	0.063	0.190
Stool consistency ^p^	0.191	0.344
Stool colour ^p^	0.305	0.457
Stool amount ^p^	0.486	0.546
Formula volume consumed ^k^	0.433	0.546
Stool frequency (day 12–14) ^k^	0.172	0.344

Analysis based on Pearson's chi-squared tests (p) or Kruskal–Wallis rank sum tests (k), *FDR* false discovery rate

The three groups, determined by hierarchical clustering, are indicated by gray ovals. The shapes indicate the sexes male (circle) or female (triangle), while the colours indicate absence (blue) or presence (red) of prior breastfeeding before the start of the trial.

Consistent with the absence of differences in microbiota composition between interventions, none of the metabolic pathways were differentially abundant upon milk-fat and vegetable-fat based IF intake. A heat map with the z-score normalized abundances of each of the found metabolic pathways over all samples is provided in Additional file 3.

### Stool fatty acids, fatty acid soaps and calcium concentration

**Fig. 4:**
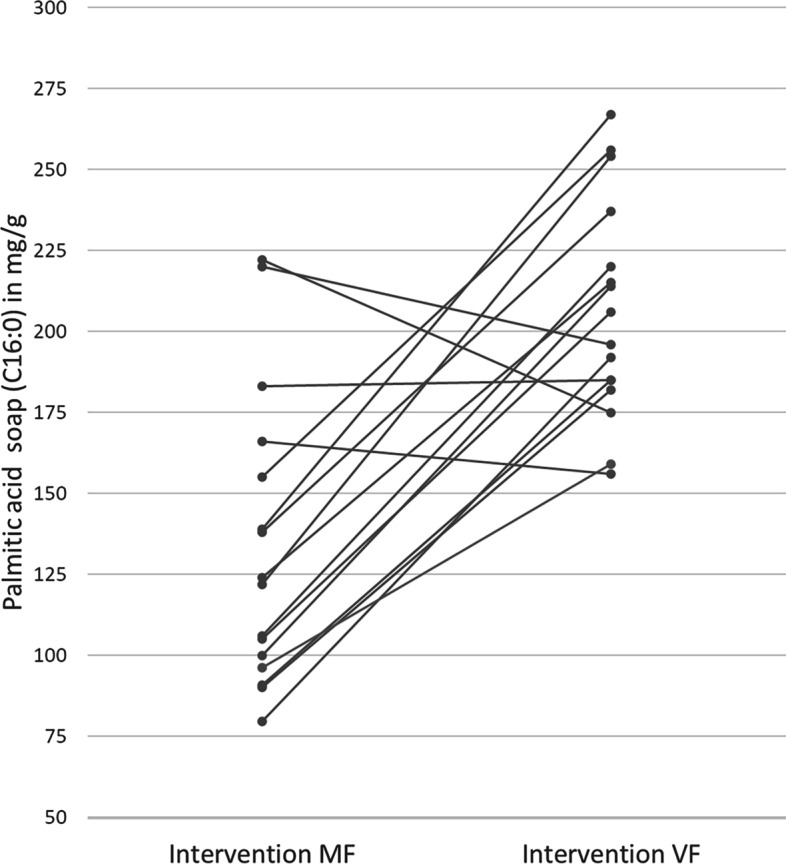
Individual palmitic acid soap values by intervention in milligrams palmitic acid soap per gram faeces. *VF* Standard formula with 100% vegetable fat source, *MF* test formula with 50% milk fat

The results of the main biochemical measurements of stool samples are shown in Table 4. The IF used in the current study strongly influenced the formation of faecal palmitic acid soap and total fatty acid soap concentrations. The MF formula with a higher level of sn2-palmitate resulted in significantly lower faecal levels of palmitic acid soap (*p* = 0.0002) and total fatty acid soaps (*p* = 0.0001) than the VF formula, as illustrated in Fig. 4 for palmitic acid soap. Concomitantly, calcium excretion and palmitic acid concentration were significantly (*p* = 0.0335)) reduced in stool samples after the MF intervention. No significant difference was noted for the total free fatty acid concentration. For faecal palmitic acid soap, the concentration as well as the proportion palmitic acid soap of total faecal soaps were significantly different between groups.

**Table 4:** Mean stool fatty acids, fatty acid soaps and calcium composition (mg/g stool dry weight)

Variable	Run-in VF (*n* = 16)	Intervention MF (*n* = 16)	Intervention VF (*n* = 16)	*p*-value intervention
Palmitic acid soap (SD), mg/g	214.3 (36.6)	133.5 (44.9)	206.2 (33.9)	**0.0002**
Palmitic acid soap (SD), %	74.2 (3.4)	58.9 (9.5)	73.4 (6.6)	**0.0003**
Palmitic acid (SD), mg/g	9.7 (13.1)	3.8 (4.6)	5.2 (6.3)	**0.0335**
Palmitic acid (SD), %	34.3 (12.4)	26.3 (12.1)	29.1 (12.7)	0.3409
Calcium excretion (SD), mg/g	47.4 (6.7)	44.4 (7.9)	47.7 (6.5)	**0.0335**
Total fatty acid soaps (SD), mg/g	288.6 (47.1)	222.7 (47.3)	281.9 (43.8)	**0.0001**
Total free fatty acids (SD), mg/g	24.1 (24.8)	14.4 (12.5)	16.4 (10.3)	0.3016

*VF* standard formula with 100% vegetable fat source, *MF* test formula with 50% milk fat, *SD* standard deviation

### Stool consistency and gut comfort

**Fig. 5:**
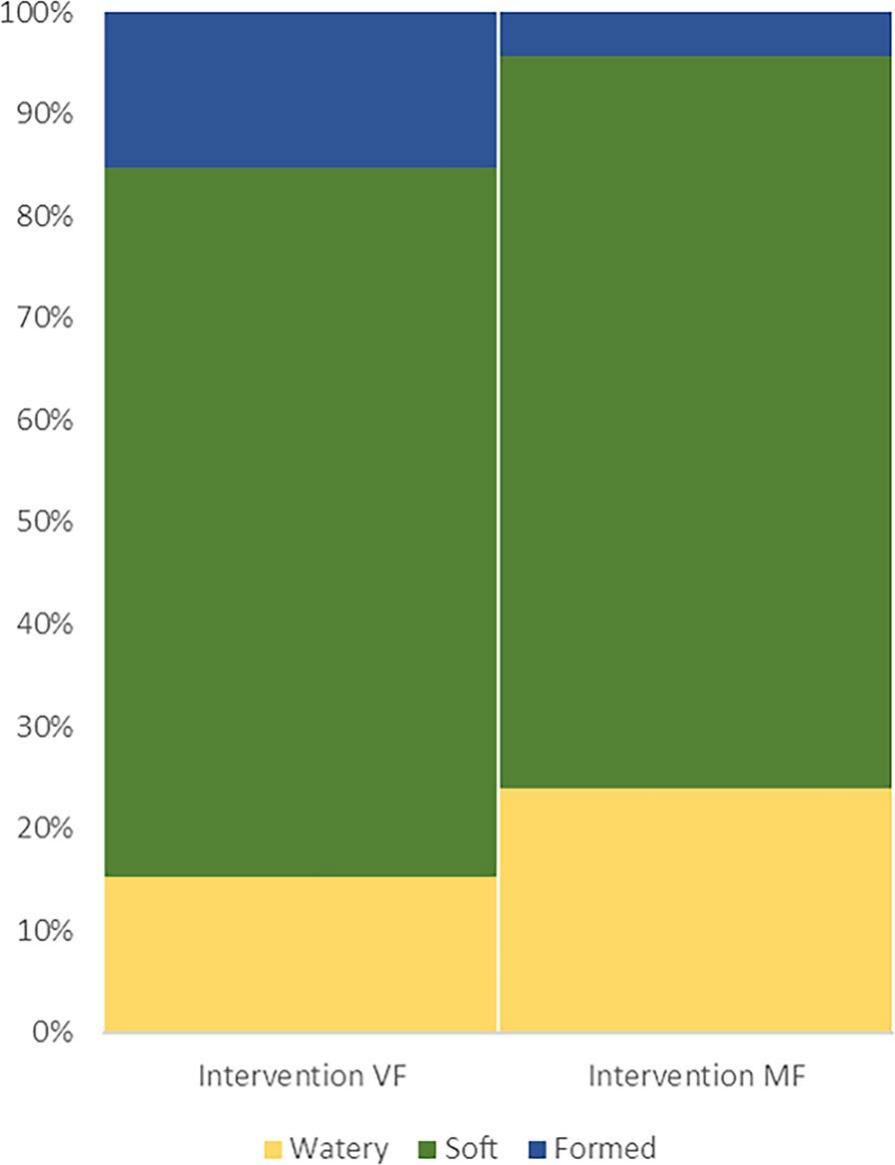
Distribution of the stool consistency categories of the Amsterdam Infant Stool Scale scores per intervention. *VF* standard formula with 100% vegetable fat source, *MF* test formula with 50% milk fat

Figure 5 shows the distribution of the ordinal stool consistency scores for the VF and MF interventions. The stool consistency scores, according to AISS, showed a *p*-value of 0.02 for the intervention fixed effect test, where the odds ratio for intervention difference was 4.0 (95% CL: 1.2–13.2). This implies that stool samples generally have a lower rank on the (ordinal) AISS scale in the MF intervention, i.e. altering the stool composition towards softer stools. AISS assigns one of four categories to an infants’ stool: Watery, Soft, Formed and Hard. The latter category, however, was not assigned to any faecal sample.

There were no significant differences for vomiting, regurgitation, colic, constipation, diarrhoea and crying episodes between interventions based on the QPGS-RIII infant/toddler (Additional file 2, Supplementary Table 3). The median stool volume by day and by period can be seen in Additional file 2, Supplementary Tables 4 and 5, respectively. No differences in stool volume were observed between the two groups as assessed by the AISS. No differences were observed between the two groups in the stool colour either (Additional file 2, Supplementary Tables 6 and 7). Most subjects had stool colour assessments of the first category of the AISS, which is yellow.

### Metabolite analysis

**Fig. 6:**
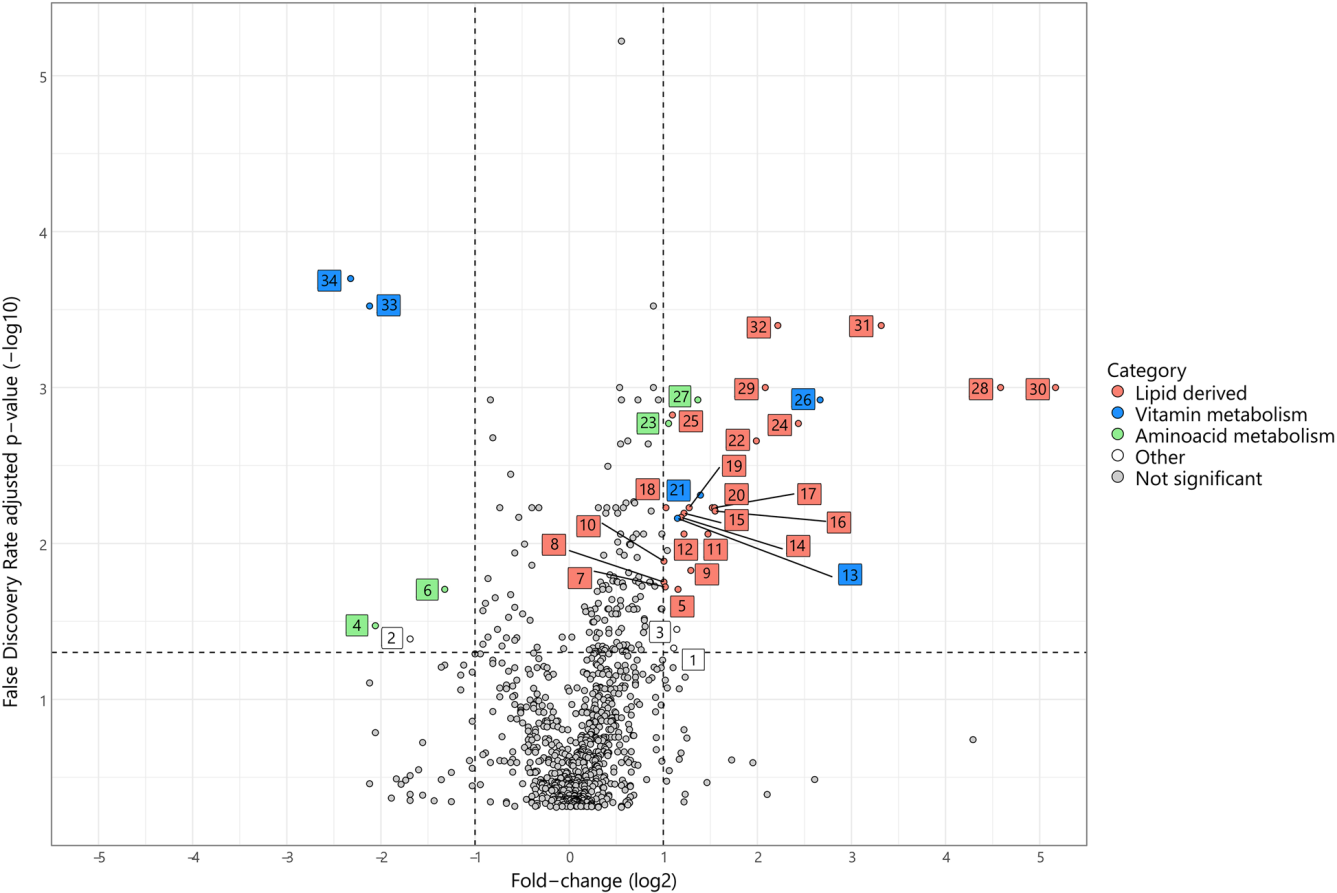
Volcano plot of measured faecal metabolites. Coloured dots indicate significantly different (FDR corrected *p* < 0.05) metabolites with at least a two-fold change between MF and VF intervention (upper left corner: higher in VF intervention; upper right corner: higher in MF intervention). 1. glutamine conjugate of C7H12O2; 2. hippurate; 3. 3-indoleglyoxylic acid; 4. gamma-aminobutyrate; 5. N-stearoylserine; 6. phenethylamine; 7. linoleoyl ethanolamide; 8. erucate; 9. docosadienoate; 10. linolenoyl ethanolamide; 11. 11-ketoetiocholanolone sulfate; 12. N-myristoyl-sphingadienine; 13. delta-tocopherol; 14. docosapentaenoate; 15. nonadecanoate; 16. (16 or 17)-methylstearate; 17. N-stearoyltaurine; 18. N-myristoyltaurine; 19. palmitoleate; 20. margarate; 21. gamma-tocopherol/beta-tocopherol; 22. (12 or 13)-methylmyristate; 23. 3-aminoisobutyrate; 24. 5-dodecenoate; 25. 3-carboxy-4-methyl-5-pentyl-2-furanpropionate; 26. carotene diol; 27. N-methylproline; 28. phytanate; 29. margaroylcarnitine; 30. pristanate; 31. (14 or 15)-methylpalmitate; 32. pentadecanoate; 33. gamma-tocotrienol; 34. alpha-tocotrienol

In total, 870 metabolites were measured in the faecal samples (see Additional file 4), of which 190 were significantly different between the MF and VF interventions determined by a crossover ANOVA (FDR corrected *p* < 0.05). The majority of these 190 metabolites were found to be higher in the faeces following MF intervention, potentially indicative of the complex composition of milk fat [19]. The 34 significantly affected metabolites with at least a twofold change between interventions are indicated in Fig. 6. Most of these are lipid-derived, but some are metabolites from vitamin or amino acid metabolism. Higher levels of faecal metabolites after MF intervention include molecules of known milk fat origin: margarate, 12/13-methylmyristate, pentadecanoate, pristanate, phytanate, docosapentaenoate, 14/15-methylpalmitate, docosadienoate, palmitoleate and carotene diol. Furthermore, several other lipid-derived molecules 3-aminoisobutyrate, 3-indoleglyoxylic acid, N-methylproline and several forms of vitamin E (tocopherols), were found at levels that were at least twofold higher after MF intervention than after VF intervention. In contrast, significantly influenced metabolites higher after VF intervention included tocotrienols, which are other forms of vitamin E. The different forms of vitamin E measured in faeces after either MF or VF intervention are consistent with the composition of the IF consumed, as tocotrienols are more abundant in the vegetable fat blend used for the VF formula than tocopherols. Hippurate, which is related to polyphenol metabolism was also higher after VF intervention, which could result from the higher levels of polyphenols in the vegetable fat blend (high in palm oil) included in the VF formula. For other metabolites higher after VF intervention (gamma-amino butyric acid (GABA) and phenethylamine), no direct link with the compositions of the study products is suspected, and these metabolites might thus reflect a treatment effect.

Random forest analysis (Additional file 2, Supplementary Fig. 3) shows that the two intervention groups can be distinguished from their metabolite profile with a predictive accuracy of 91%. In addition, it provides the metabolites that are most predictive of the type of intervention. These metabolites partly overlap with the metabolites shown in Fig. 6, but in addition several carnitines, ergosterol, 7-ketocholesterol, lyxonate, myristate and laurate helped to distinguish both groups.

## Discussion

### Faecal palmitate soap and stool consistency

The current study demonstrates that milk fat-based IF reduces faecal palmitate soap and total fatty acid soap formation as well as calcium excretion in healthy term infants compared to a fully vegetable fat-based IF with a lower level of sn-2 palmitate. These results confirm the findings of a previous trial with identical IF products [5] and are consistent with trials using structured vegetable fat blends high in sn-2 palmitate [8, 20]. Milk fat is a natural source of sn-2 palmitate. In addition to lowering faecal fatty acid soaps, inclusion of milk fat in IF delivers beneficial compounds such as cholesterol, phospholipids, and sphingolipids [2]. The milk fat-based formula in our study positively influenced stool consistency, a finding in line with previous results [5]. This is most likely a direct effect of reduced faecal fatty acid soaps. Gut microbiota composition could also play a role since significant associations have been found between stool consistency and microbial richness in adults [21, 22]. The gut bacteria might be stimulated by specific fatty acids or be inhibited by fatty acid soaps.

### Gut microbiota

Higher faecal levels of beneficial bacteria such as bifidobacteria and lactobacilli have been shown in faeces of infants fed high sn-2 palmitate diets compared to infants on low sn-2 palmitate diets [7–9] at dosages that were comparable with those in our study. Furthermore, a recent in vitro study demonstrated growth inhibition of several beneficial bacteria, including strains of bifidobacteria, by calcium palmitate [6]. In our study, no significant difference was found for bifidobacteria between the MF and VF interventions. An explanation might be the presence of galactooligosaccharides (GOS) in both formulae at levels that are used in commercial IF. GOS are prebiotic components known to stimulate the growth of bifidobacteria and lactobacilli [23, 24]. The prebiotic effect of GOS could have overshadowed any growth-inhibiting effect of faecal fatty acid soaps. GOS was also used in the study of Wu et al. [9] but at a lower concentration. After the run-in period in which the infants consumed the standard VF formula with GOS, the median relative abundance of bifidobacteria was 60% in our study. This left little room for further improvement. The median relative abundance of bifidobacteria at baseline in the study of Wu et al. [9] was below 10% and increased to 15% after high sn-2 palmitate intervention. Presumably, oligosaccharides are more potent drivers of bifidobacteria than specific fat structures, such as beta palmitate. Another explanation could be the relatively low number of subjects in our study. However, in the study of Yaron et al. [7], the number of subjects was comparable, and positive effects on bifidobacteria were found using IF high in sn-2 palmitate. The infant formula in their study did not contain any prebiotics, which makes the explanation of not finding an effect on bifidobacteria in the current study due to the GOS effect more likely than the number of subjects. A third explanation may be the age of the infants. In Yaron, Wu and Yao [7–9], the infants were within 14 days of age at inclusion, whereas in the current study, the infants were 3 months of age at inclusion.

Other bacterial taxa were also not influenced by the intervention. The limited effect of bovine milk fat on gut microbiota composition is in line with results of studies with milk fat globule membrane (MFGM) that showed only moderate effects on gut microbiota composition [25, 26].

Beta-diversity analysis of microbiota composition separated the faecal samples into three different groups that were not related to the intervention. Important clinical parameters associated with the group separation were prior breastfeeding and gender. The lasting effects of breastfeeding on gut microbiota composition are well known [27]. The relationship between gender and microbiota composition has not been thoroughly studied, although gender differences in microbiota have been observed in several studies [28, 29]. Despite the known effect of mode of birth on gut microbiota composition, we did not find an association of the clusters with mode of birth. We did not perform measurements before the run-in period, but it is likely that the differences in gut microbiota composition detected by beta-diversity analysis were already present before the start of the trial.

### Faecal metabolites

Despite the absence of apparent effects of the study formulae on microbiota composition, 21.8% of the 870 faecal metabolites measured were significantly different between the VF and MF interventions (FDR corrected *p-*value < 0.05). Several metabolites with the highest fold changes reflected differences in concentrations of these molecules in the formulae, such as milk fat-specific fatty acids and different forms of vitamin E. The odd chain fatty acids pentadecanoate (C15) and margarate (C17) have been used in various studies as serum and adipose tissue markers of dairy fat intake [30]. In agreement with that, our results demonstrate higher levels of these markers in faeces after MF intervention. Branched chain fatty acids (BCFAs) found elevated after MF intervention, such as 14/15-methylpalmitate, 12/13-methylmyristate, pristanate and phytanate, are also directly derived from milk fat [31]. Increased faecal levels of BCFAs could be beneficial, as based on in vitro work, it is assumed that BCFAs are important for the growth and metabolism of enterocytes [32] and reduce inflammatory markers [33]. Additionally, palmitoleate, another milk fat-specific fatty acid that increased after MF intervention, may be associated with several health benefits, such as favourable metabolic health outcomes [34].

Furthermore a difference between interventions was found for hippurate, a microbial metabolite of polyphenolic compounds. This can be explained by the levels of polyphenols in palm oil [35], which formed a significant part of the vegetable fat source of the VF formula. In addition, differences were detected in the levels of microbial compounds that could not be linked to the composition, like GABA. GABA is an important inhibitory neurotransmitter that can be produced by several bacteria, such as *Bifidobacterium*, *Bacteroides* and *Escherichia* species [36, 37], and can also be consumed by several gut bacteria [36]. GABA levels were significantly lower in the MF intervention, but we could not detect a correlation between specific microbial species and faecal GABA levels. This is in contrast to the study of Altaib et al. [37] that showed significant separation between samples high and low in GABA through beta-diversity analysis of the microbiota. In our MDS plots, samples of subjects clustered together despite large differences in GABA content. Although we did not find a relation between GABA levels and specific microbial species or overall microbiota composition, we cannot exclude the possibility that the difference in GABA is caused by microbial activity. GABA synthesis is highly strain dependent [38], and differences in strain content were not determined. However, there was no difference in abundance of the pathway for GABA synthesis between interventions. Hypothetically, differences might also be caused by a difference in absorption of GABA from the gut, but as no serum levels of this metabolite were measured, this remains inconclusive and requires further investigation.

We did not include a breast milk-fed control group in our study. We do, however, expect that the metabolic profile of breastfed infants is more similar to that after MF intervention than after VF intervention, as bovine milk fat more closely resembles human milk fat than vegetable fat blends [2]. To the best of our knowledge, studies directly comparing faecal metabolites of breastfed infants and milk fat-based formula-fed infants have not yet been reported.

## Conclusions

In summary, this study confirms the beneficial effects of replacing vegetable fat with milk fat in infant formula by improved stool consistency and lower formation of faecal fatty acid soaps. However, no significant differences in stool microbiota composition were observed between the nutritional interventions, possibly due to the presence of GOS in both formulae. In addition, metabolites were significantly different between the intervention groups. Whether these differences are merely the representation of the different fat blends and/or drive the clinical differences remains to be explored.

## Supplementary Information

**Additional file 1.** Methods to determine QPGS-RIII, stool consistency, volume and colour

**Additional file 2.** Supplementary tables and figures

**Additional file 3.** Heatmap normalized pathway analysis

**Additional file 4.** Raw data metabolomics

## Data Availability

The dataset supporting the conclusions of this article is available in the Bioproject repository, PRJNA798191, https://www.ncbi.nlm.nih.gov/bioproject/798191. The trial protocol is available from the corresponding author upon reasonable request.

## Abbreviations

AE: Adverse events
AISS: Amsterdam Infant Stool Scale
BCFAs: Branched chain fatty acids
BF: Breastfed
C16:0: Palmictic acid
FDR: False discovery rate
FF: Formula-fed
GABA: Gamma-amino butyric acid
GOS: Galacto-oligosaccharides
HM: Human milk
HMO: Human milk oligosaccharides
IF: Infant formula
LCSFA: Long chain saturated fatty acid
MDA: Mean decrease accuracy
MF: Milk fat-based formula with 50% milk fat
PA: Palmitic acid
RFA: Random forest analysis
SAE: Serious adverse events
TAG: Triacylglycerol
VF: Vegetable fat-based formula with 100% vegetable fat source
QPGS-RIII: Questionnaire on Paediatric Gastrointestinal Symptoms-Rome III
