# Supplementary material for: Effect of bovine milk fat-based infant formulae on microbiota, metabolites and stool parameters in healthy term infants in a randomized, crossover, placebo-controlled trial
Source: BMC Nutr. 2022 Aug 29;8:93. doi: 10.1186/s40795-022-00575-y (PMC9426040; doi:10.1186/s40795-022-00575-y)
Supplement: Supplementary file 1 — Additional file 1. Methods to determine QPGS-RIII, stool consistency, volume and colour [file 40795_2022_575_MOESM1_ESM.docx]

This supplementary data are part of the Bamboo clinical trial which was registered at <https://www.trialregister.nl/trial/7815> [Netherlands Trial Register Identifier: Trial NL7815].

Additional file 1

**Methods to determine QPGS-RIII, stool consistency, volume and colour**.

QPGS-RIII infant/toddler questionnaire consists of seven sections for different symptoms. Each section has first a general question about the symptom, e.g. “Has your child spit-up or vomited two or more times daily in the past two weeks” with a dichotomous response (yes/no). Parents who agree with the question are instructed to complete the other questions in that subsection; parents who do not agree are instructed to move to the next section. Individual questions were summarized descriptively with subject counts and percentages. Subsection answer category percentages were calculated related to the number of subjects that completed the subsection questions. Difference between treatments for the proportion of answers (yes/no) for each of the section general questions were tested using McNemar’s test.

AISS was used to evaluate and compare stool characteristics (consistency, volume, colour) between the Milk Fat and vegetable fat formula. Stool volume and stool colour were evaluated as ordered categorical variables. Observations were summarized using descriptive statistics by observation day and treatment (counts and percentages). In addition, the overall treatment period was summarized tabulating the number and proportion (%) of subjects and number of categories. For analysis purposes stool colour was ordered from dark to light. For statistical inference, new variables were derived for volume and colour, where the value took the median of the original variable observations within the treatment period and day. In case of ties, the latest value (timewise) was selected. Descriptive statistics (counts and percentages) were tabulated by day and treatment. Repeated measures ordinal regression analysis (using cumulative logits) taking account the paired nature of the observations from different treatments was performed to formally compare the treatments.
