# Supplementary material for: Effect of bovine milk fat-based infant formulae on microbiota, metabolites and stool parameters in healthy term infants in a randomized, crossover, placebo-controlled trial
Source: BMC Nutr. 2022 Aug 29;8:93. doi: 10.1186/s40795-022-00575-y (PMC9426040; doi:10.1186/s40795-022-00575-y)
Supplement: Supplementary file 2 — Additional file 2. Supplementary tables and figures [file 40795_2022_575_MOESM2_ESM.pdf]

This supplementary data are part of the Bamboo clinical trial which was registered at https://www.trialregister.nl/trial/7815 [Netherlands Trial Register Identifier: Trial NL7815].

Additional file 2 Supplementary Tables and Figures.

Supplementary Table 1 Data per subject

			Available data			
**Subject**	intervention period 1	intervention period 2	microbiota	metabolites	fatty acid profile & calcium	questionnaires
**1**	MF	VF	Y	Y	Y	Y
**2**	MF	VF	Y	Y	Y	Y
**3**	MF	VF	Y	Y	Y	Y
**4**	MF	VF	Y	Y	Y	Y
**5**	MF	VF	Y	Y	Y	Y
**6**	VF	MF	**N**	**N**	**N**	Y
**7**	VF	MF	Y	Y	Y	Y
**8**	VF	MF	Y	Y	Y	Y
**9**	MF	VF	**N**	**N**	**N**	Y
**10**	VF	MF	Y	Y	Y	Y
**11**	VF	MF	Y	Y	Y	Y
**12**	VF	MF	Y	Y	Y	Y
**13**	MF	VF	Y	Y*	Y	Y
**14**	MF	VF	Y	Y	Y	Y
**15**	MF	VF	Y	Y	Y	Y
**16**	VF	MF	Y	Y	Y	Y
**17**	VF	MF	Y	Y	**N**	**N**
**18**	VF	MF	Y	Y	Y	Y
**19**	MF	VF	Y	Y	Y	Y
			n=17	n=17	n=16	n=18

* except for second time point;
VF: standard formula with 100% vegetable fat source; MF: test formula with 50% milk fat; Y: data available; N: data not available. MF-VF: crossover group with MF first; VF-MF: crossover group with VF first

Supplementary Table 2 Formula consumption and anthropometric data.

	**Run-in VF (n=19)**	**Intervention MF (n=18)**	**Intervention VF (n=19)**	***p*-value intervention**
**Average weekly milk intake, ml**				0.243
Mean (SD)	5907 (763)	5966 (1155)	6317 (1132)	
Median	6125	5985	6139	
Min-max	4284-7070	4200-8729	4704-8589	
**Weight, g**				
Mean (SD)	6380 (746)	6819 (817)	6877 (864)	0.799
Median	6450	6652	6907	
Min-max	4900-7500	5600-8540	5050-8197	
**Length, cm**				
Mean (SD)	64.1 (2.5)	66.0 (2.5)	66.3 (3.0)	0.890
Median	64.0	65.4	65.8	
Min-max	59.9-70.0	61.0-70.0	61.5-71.9	

Comparisons between the formula groups were conducted using the Wilcoxon signed-rank test; VF: standard formula with 100% vegetable fat source; MF: test formula with 50% milk fat.

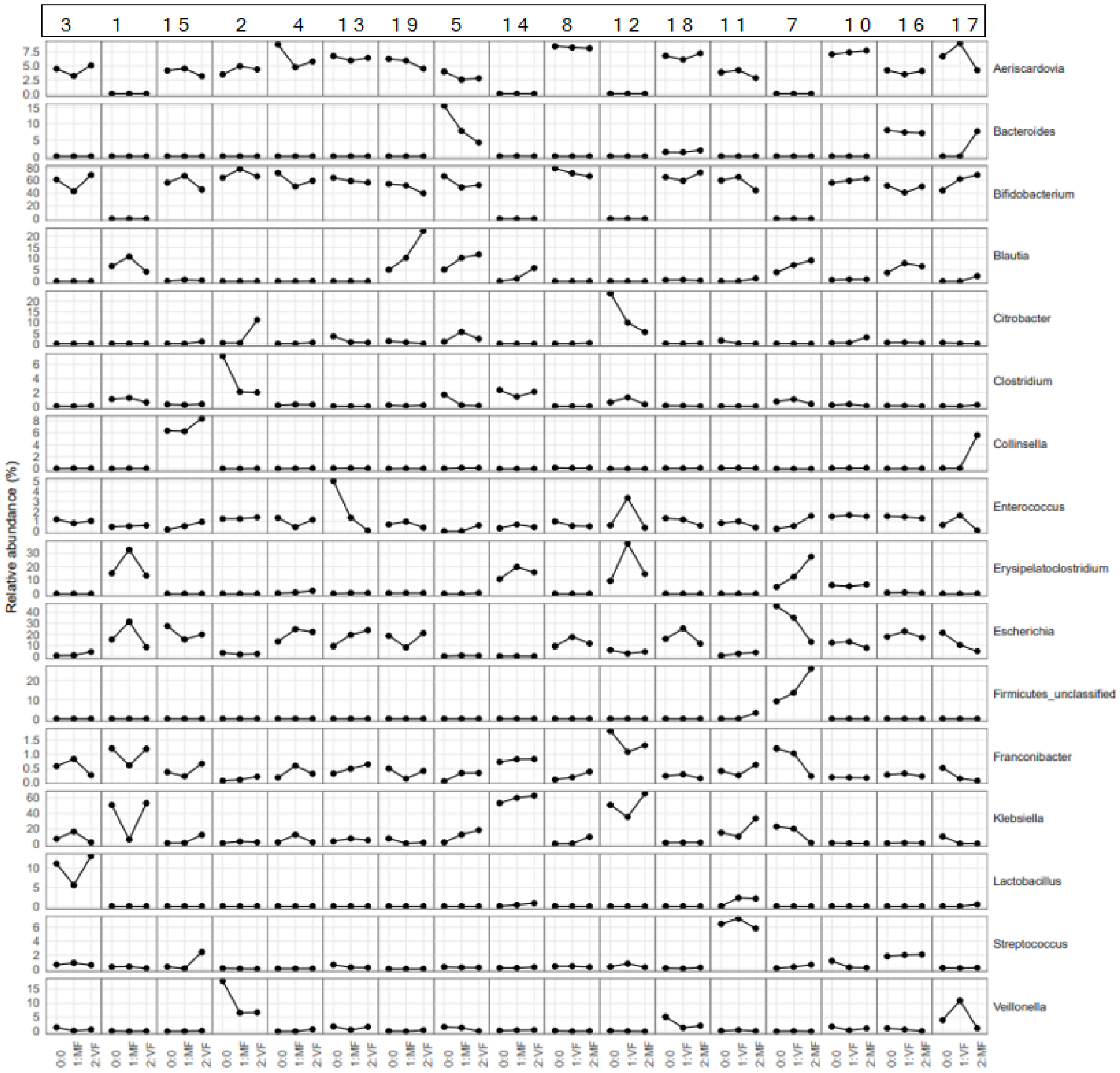

Supplementary Fig 1 Relative abundances of the 16 most abundant genera in each of the three faecal samples of the subjects.

The first type of intervention given followed by the subject number is indicated per subject at the top of the figure. 0: baseline; VF: standard formula with 100% vegetable fat source; MF: test formula with 50% milk fat

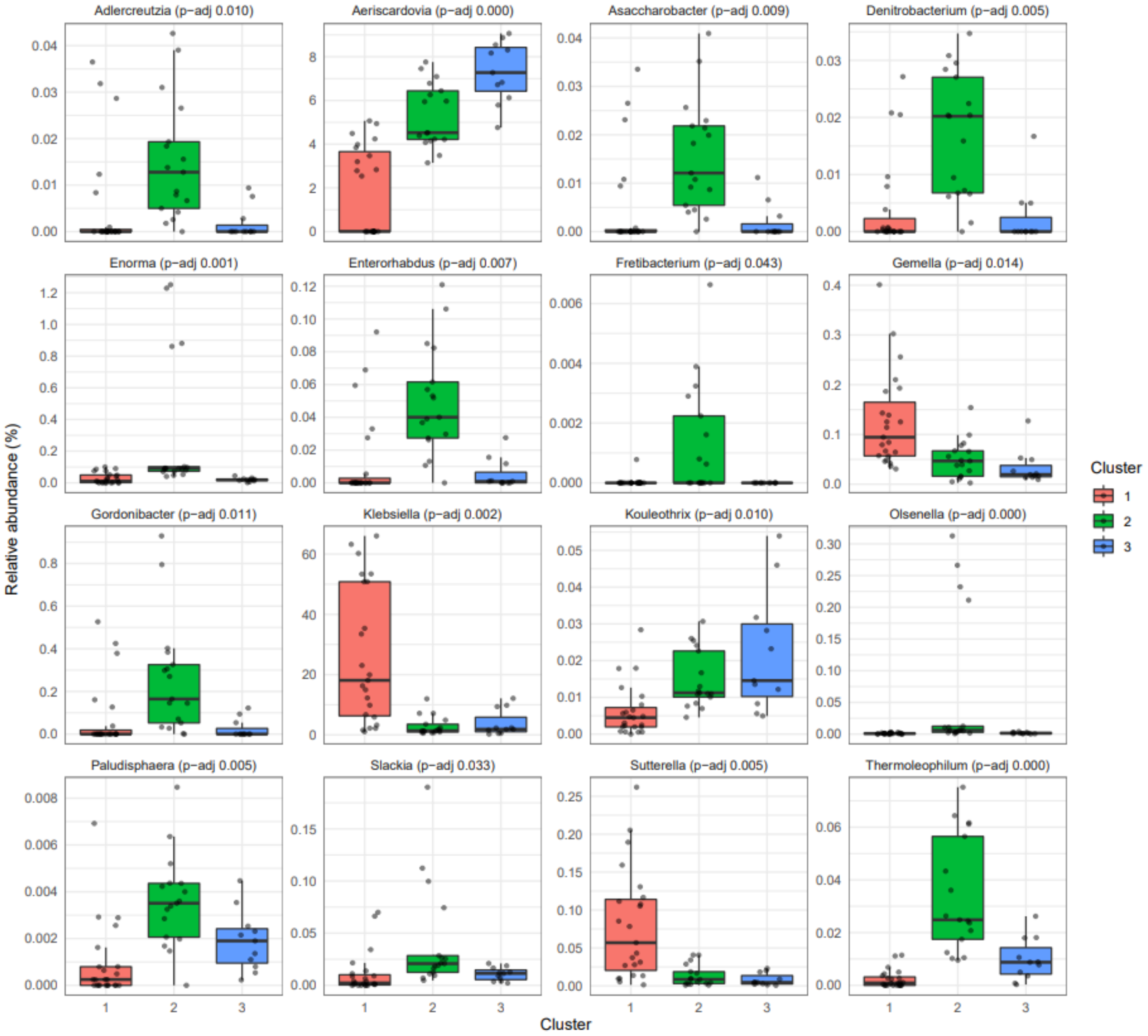

Supplementary Figure 2 Differences in microbiota composition between clusters.

Boxplots of the relative abundances per cluster of the 16 genera that were significantly different between the 3 groups that are shown in figure 3 of the paper.

Supplementary Table 3 QPQS-RIII infant/toddler statistical analysis.

**Section/Question**	***p*-value intervention (MF vs. VF formula)**
Section A: Vomiting	0.6875
Section B: Bring up food	0.1250
Section C: Crying	1.0000
Section D: Hurt when pooping	1.0000
Section E: Pooping difficulties with soft stool	0.1250

McNemar's test for QPGS-RIII Infant/toddler questionnaire by section, main level of each section analyzed; VF: standard formula with 100% vegetable fat source; MF: test formula with 50% milk fat.

Supplementary Table 4 Stool sample's volume median for day.

	Day	Class	Run in VF (N=18)	Intervention MF (N=18)	Intervention VF (N=18)	Difference between groups (MF vs. VF formula) OR	95% CI	*p*-value
Stool volume, n (%)	12	Smear		5 (31.3)		1.016	0.461, 2.237	0.9685
		Up to 25%	2 (11.1)	3 (18.8)	6 (35.3)			
		25-50%	8 (44.4)	5 (31.3)	6 (35.3)			
		>50%	8 (44.4)	3 (18.8)	5 (29.4)			
	13	Smear	1 (6.3)	3 (20.0)	3 (20.0)			
		Up to 25%	2 (12.5)	4 (26.7)	2 (13.3)			
		25-50%	6 (37.5)	4 (26.7)	7 (46.7)			
		>50%	7 (43.8)	4 (26.7)	3 (20.0)			
	14	Smear	2 (16.7)	2 (13.3)	5 (35.7)			
		Up to 25%	1 (8.3)	1 (6.7)	3 (21.4)			
		25-50%	6 (50.0)	8 (53.3)	5 (35.7)			
		>50%	3 (25.0)	4 (26.7)	1 (7.1)			

Repeated measures cumulative logit-model, modelling the probability of stool volume having lower ordered values.

VF: standard formula with 100% vegetable fat source; MF: test formula with 50% milk fat. OR: odds ratio; CI: confidence interval.

Supplementary Table 5 Stool sample's volume, median for period.

	Class	Run in VF (N=18)	Intervention MF (N=18)	Intervention VF (N=18)
Stool volume, n (%)	Smear	1 (5.6)	2 (11.1)	3 (16.7)
	Up to 25%	2 (11.1)	4 (22.2)	4 (22.2)
	25-50%	7 (38.9)	8 (44.4)	9 (50.0)
	>50%	8 (44.4)	4 (22.2)	2 (11.1)

VF: standard formula with 100% vegetable fat source; MF: test formula with 50% milk fat.

Supplementary Table 6 Stool sample's colour, median for day.

	Day	Class	Run in VF (N=18)	Intervention MF (N=18)	Intervention VF (N=18)	Difference between groups (MF vs. VF formula) OR	95% CI	*p*-value
Stool colour, n (%)	12	I	15 (83.3)	7 (43.8)	13 (76.5)	0.402	0.142, 1.137	0.0848
		II	1 (5.6)	5 (31.3)	2 (11.8)			
		III	2 (11.1)	3 (18.8)	2 (11.8)			
		VI		1 (6.3)				
	13	I	11 (68.8)	7 (46.7)	10 (66.7)			
		II	2 (12.5)	4 (26.7)	4 (26.7)			
		III	2 (12.5)	3 (20.0)				
		IV		1 (6.7)				
		VI	1 (6.3)		1 (6.7)			
	14	I	10 (83.3)	12 (80.0)	8 (57.1)			
		II	1 (8.3)	2(13.3)	5 (35.7)			
		III		1 (6.7)	1 (7.1)			
		IV	1 (8.3)					

Repeated measures cumulative logit-model, modelling the probability of stool colour having lower ordered (lighter) values. VF: standard formula with 100% vegetable fat source; MF: test formula with 50% milk fat. OR: odds ratio; CI: confidence interval.

Supplementary Table 7 Stool sample's colour, median for period.

	Class	Run in VF (N=18)	Intervention MF (N=18)	Intervention VF (N=18)
Stool colour, n (%)	I	14 (77.8)	10 (55.6)	11 (61.1)
	II	2 (11.1)	5 (27.8)	5 (27.8)
	III	2 (11.1)	3 (16.7)	2 (11.1)

VF: standard formula with 100% vegetable fat source; MF: test formula with 50% milk fat

A.

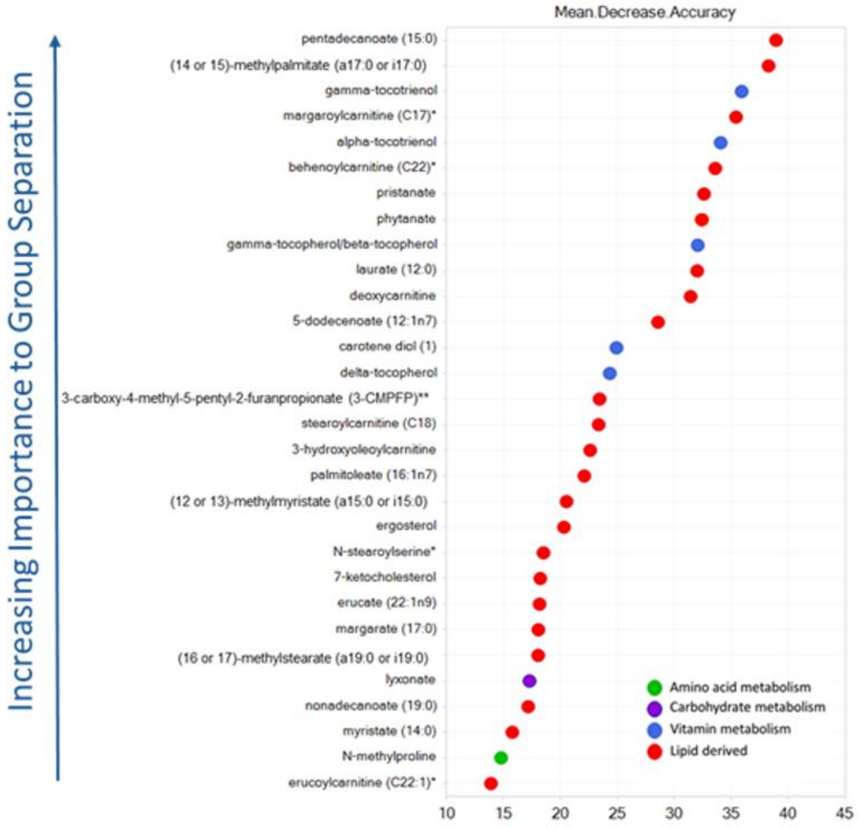

B.

Random Forest Confusion Matrix

Predicted Group

Actual Group

Feces	MF	VF	Class Error
MF	13	3	18.75
VF	0	17	0.00
Predictive accuracy = 90.91%			

C.

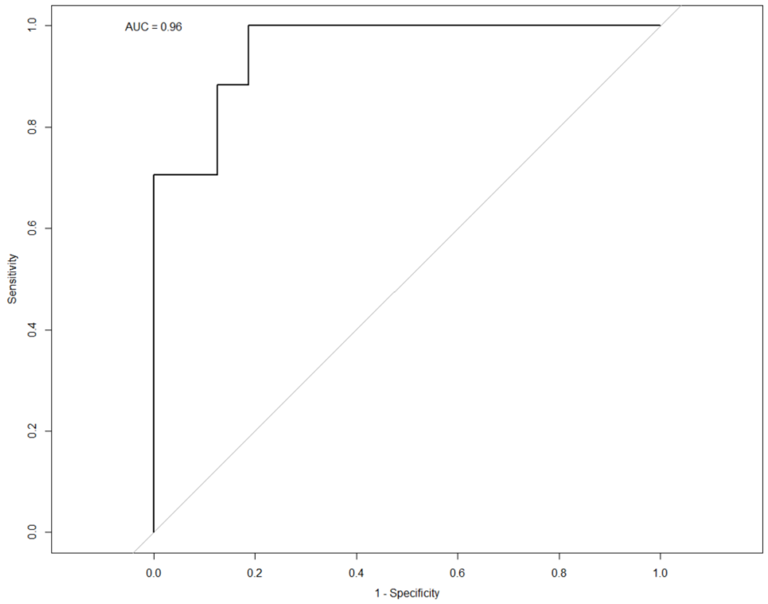

Supplementary Figure 3 Random Forest Analysis.

A. Metabolites that made the largest contribution to the separation of samples based on intervention.
B. Random forest confusion matrix
C. Receiver operating characteristic (ROC) curve and AUC for the Random Forest Analysis
VF: standard formula with 100% vegetable fat source; MF: test formula with 50% milk fat
