# Supplementary figures and images for: Effect of bovine milk fat-based infant formulae on microbiota, metabolites and stool parameters in healthy term infants in a randomized, crossover, placebo-controlled trial
Source: BMC Nutr. 2022 Aug 29;8:93. doi: 10.1186/s40795-022-00575-y (PMC9426040; doi:10.1186/s40795-022-00575-y)

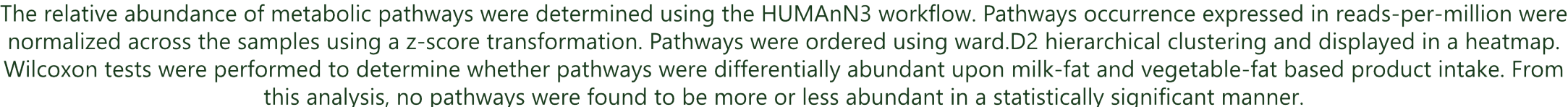

Supplement: Supplementary file 3 — Additional file 3. Heatmap normalized pathway analysis [file 40795_2022_575_MOESM3_ESM.pdf]
